# Editorial: Influence of Inter- and Intra-Synaptic Factors on Information Processing in the Brain

**DOI:** 10.3389/fncom.2019.00032

**Published:** 2019-05-21

**Authors:** Vito Di Maio, Jean-Marie C. Bouteiller

**Affiliations:** ^1^Istituto di Scienze Applicate e Sistemi Intelligenti (ISASI) del CNR, Pozzuoli, Italy; ^2^Department of Biomedical Engineering, University of Southern California, Los Angeles, CA, United States

**Keywords:** synaptic transmission, dendritic integration, synaptic plasticity, neural modeling, neural network, glutamatergic, GABAergic

The main function of the brain is to process and integrate information coming from the environment and from other parts of the body and produce appropriate responses.This information processing capability is mimicked in neurons in which dendritic arborization integrates information from thousands of synapses, both excitatory and inhibitory. The main activity of a neuron then consists in receiving synaptic inputs and integrate them to produce spike sequences (neural code), which ultimately form the presynaptic inputs to other neurons. Evidently, synaptic transmission represents an essential building block of information processing in the brain.

Synapses show large variability in the amplitude, time duration, and time-dependent probability of their produced output as a function of a presynaptic stimulus. Partially, this variability can be assumed to be of stochastic origin (e.g., position of the vesicles at the presynaptic side), but the larger part is due to the fine regulation of synaptic components, e.g., the activity-dependent regulation of the number of postsynaptic receptors (through long-term potentiation and long-term depression), their sub-unit composition and the biophysical properties of the neuronal area where they are located.

These regulatory mechanisms determine the intensity, frequency, ability, and quality of the information transmitted; they, in addition to the biophysical properties of both pre and post-synaptic neurons are responsible for shaping the whole neural activity.

Unsurprisingly, these mechanisms and their effects on neuronal activity in both physiological and pathological cases have been intensely studied. Yet a lot remains to be learned and achieving a better understanding of the mechanisms involved is still of paramount importance.

The present research topic aims to collect papers by authors who, using approaches of both experimental and computational nature, strengthen our understanding of synaptic function and its role in information processing. This collection provides a non-exhaustive yet multiscale perspective on synapses, ranging from the molecular contribution of proteins on the regulation of synaptic transmission, to the study of the effects of synaptic activity on network-level activity.

A molecular switch involving the protein CaMKII in short period plasticity is proposed by Clarke, who analyses the bistable properties of this molecule driven by Ca^2+^ cytosolic transients using a computational approach.

To study stochastic short-term plasticity, a method based on hidden Markov models (HMMs) is proposed by Mokhtari et al.

Guerrier and Holcman present a review of the intricate interactions that take place in the presynaptic terminal leading to the release of neurotransmitters; the presynaptic sensors for Ca^2+^ are considered for their role in the probability of vesicle release and the contribution to plasticity phenomena.

All synaptic inputs are not equal and they strongly depend on the location of the synapses on the dendritic tree. Felton et al. present a study of postsynaptic integration and the interactions between multiple functional zones. They use stochastic resonance models to perform their analysis and demonstrate the presence of meaningful interactions between these different functional zones.

GABA is known to mediate intercellular communications by participating in both “wiring” and “volume” transmission. The “wiring” action of GABA takes place via synaptic transmission by activating the postsynaptic (phasic) GABAA-receptors. The “volume” transmission is carried out by “overspilled” ambient GABA which regulates neuronal excitability by creating extra transmembrane current through extrasynaptic (tonic) GABAA-receptors. In their contribution, Adamchik et al. propose a simple computational model that simulates the effects of non-stationary, activity dependent GABA upon population dynamics of interneurons and demonstrate that this effect leads to relaxation oscillations.

Ferguson and Gao present a study of the regulatory effects of inhibitory synapses on the activity of prefrontal cortical circuits neurones. They outline the role of inhibition in regulating the excitatory activity of glutamatergic neurons showing how lack of balance between excitatory and inhibitory synapses can be the base of specific pathologies.

Spike-timing dependent plasticity (STDP) is considered one of the major processes underlying memory storage and recall. Langlois et al. show the importance of dopaminergic synapses on the regulation of the GABAergic STDP, which plays a critical role in the circuitry between the deep nuclei of the brain and the cortex.

Other mechanisms have also been shown to contribute to modulation of STDP; Foncelle et al. propose a review of the different mechanisms reported to affect this modulation.

Based on a theoretical model, Millán et al. show that the pruning (and formation) of synapses alters the energy landscape of an associative network such that the neural system becomes able to track several memories or attractors by oscillation-like dynamics. They argue that this oscillation is induced by destabilization of the current attractor.

Calcium dynamics has been shown to play a critical role for normal brain function as it affects synaptic homeostasis and promotes learning and memory. Additionally, it has been implicated in pathologies, and most notably neurodegeneration and Alzheimer's disease. Hu et al. present an integrated mechanistic model of postsynaptic calcium concentration dynamics that incorporates various already published elementary models and demonstrate its accuracy using various experimental results. Notably, an important factor that impedes our understanding of the nervous system is its multiscale complexity. Building biologically accurate computational models that aim to span several scales quickly becomes impractical due to high computational load. This limitation holds true for the instantiation of many mechanistic models of calcium dynamics in simulations comprising a large number of neurons (each neuron containing thousands of synapses). To circumvent this limitation, Hu et al. also present an input-output model that accurately summarizes the functional dynamics of the mechanistic model, while significantly reducing the computational complexity, thereby enabling realistic large-scale simulations.

Visualizing and quantifying molecular interactions taking place at the synaptic level is a challenging task given the nanoscopic scale of the system, as well as the rapid temporal changes. Yet a growing number of tools now allow live recordings of various signaling pathways and protein-protein interaction dynamics in time and space by ratiometric measurements, such as Bioluminescence Resonance Energy Transfer (BRET) Imaging. Chastagnier et al. present a single-cell BRET imaging protocol that is used to visualize protein-protein interactions in living cells at subcellular level. The visualization technique, introduced by the same group in 2016, uses Nanoluciferase as a BRET donor partner in performing the imaging assay. Here, the authors present the steps needed to analyze BRET images concurrently with a toolset they have developed called ”BRET-Analyzer” that facilitates systematic quantitative analysis.

The present research topic constitutes a collection of studies that investigate open questions on synaptic processes and their effect on information processing. We trust these will add to the collective knowledge and be of interest to scientists engaged in the topic.

## Author Contributions

Both authors listed have made equal, direct and intellectual contribution to the work, and approved it for publication.

### Conflict of Interest Statement

The authors declare that the research was conducted in the absence of any commercial or financial relationships that could be construed as a potential conflict of interest.

